# Interaction of High Flash Point Electrolytes and PE-Based Separators for Li-Ion Batteries

**DOI:** 10.3390/ijms160920258

**Published:** 2015-08-27

**Authors:** Andreas Hofmann, Christoph Kaufmann, Marcus Müller, Thomas Hanemann

**Affiliations:** 1 Institut für Angewandte Materialien-Werkstoffkunde, Karlsruher Institut für Technologie (KIT), Hermann-von-Helmholtz-Platz 1, 76344 Eggenstein-Leopoldshafen, Germany; E-Mail: christoph.kaufmann1@gmx.de; 2 Institut für Angewandte Materialien-Keramische Werkstoffe und Technologien, Hermann-von-Helmholtz-Platz 1, 76344 Eggenstein-Leopoldshafen, Germany; E-Mail: marcus.mueller@kit.edu; 3 Institut für Mikrosystemtechnik, Universität Freiburg, Georges-Köhler-Allee 102, 79110 Freiburg, Germany; E-Mail: thomas.hanemann@kit.edu

**Keywords:** Li-ion battery, electrolytes, separators, contact angle, safety

## Abstract

In this study, promising electrolytes for use in Li-ion batteries are studied in terms of interacting and wetting polyethylene (PE) and particle-coated PE separators. The electrolytes are characterized according to their physicochemical properties, where the flow characteristics and the surface tension are of particular interest for electrolyte–separator interactions. The viscosity of the electrolytes is determined to be in a range of η = 4–400 mPa∙s and surface tension is finely graduated in a range of γ_L_ = 23.3–38.1 mN∙m^−1^. It is verified that the technique of drop shape analysis can only be used in a limited matter to prove the interaction, uptake and penetration of electrolytes by separators. Cell testing of Li|NMC half cells reveals that those cell results cannot be inevitably deduced from physicochemical electrolyte properties as well as contact angle analysis. On the other hand, techniques are more suitable which detect liquid penetration into the interior of the separator. It is expected that the results can help fundamental researchers as well as users of novel electrolytes in current-day Li-ion battery technologies for developing and using novel material combinations.

## 1. Introduction

All cell materials that are used in state-of-the-art Li-ion batteries are carefully developed and optimized to guarantee well-working and stable cell performance and optimal energy storage. With the development of novel electrode materials, separators or electrolytes with improved properties, the compatibility of all cell components has to be proved anew [1]. Therefore, relatively easy and readily available methods are required that can be used to preselect and screen potential material combinations and novel materials. Such techniques include optical techniques as well as physicochemical measurements. Electrolytes and separators are two of the main component units of Li-ion cells for enabling the lithium transport between both electrodes and ensuring the separation of both electrodes. Both materials are still under intense investigation to improve lithium transport, enhance temperature stability and increase the high voltage stability and internal resistance as well as the cell lifetime in order to ensure a maximum of cell safety [2,3,4,5]. These aims necessitate the substitution of selected components and chemicals.

Mono- or multi-layer polyolefin separators are one of the major classes of separator materials that are used in current Li-ion cells [2]. Particle coated olefin separators can significantly improve the cell performance and particularly the cell safety in terms of temperature stability, lithium dendrite barrier and preventing thermal runaway [6,7,8,9,10]. For this purpose, the separator surface is coated by ceramic particles, which modify the top surface polarity and form a thermostable barrier. Besides, such a procedure can improve the electrolyte–separator interaction [11] as well as the cell performance and accelerate the wetting of the separator, which is affected, among other factors, by porosity, surface roughness and electrolyte viscosity [12]. Recently, Shi *et al*. investigated a carboxymethyl cellulose based approach to significantly improve PE-based separators in terms of safety and wettability [13]. An increase in the safety of the separator can also be obtained by a polymer coating to some extent [14,15]. In industrial processes, the electrolyte filling comes along with vacuum, which improves the wetting and electrolyte uptake, too. Novel electrolytes, which improve cell safety and high voltage stability, usually entail properties such as enhanced viscosity, reduced ionic conductivity/lithium mobility and modified polarity and solubility. Typical classes of potential and promising electrolyte solvents include ionic liquids, esters, nitriles, sulfolanes, organic carbonates and mixtures thereof [1,16,17,18,19,20,21,22,23,24,25,26,27,28]. The replacement of LiPF_6_ by lithium bis (trifluoromethanesulfonyl) azanide (LiTFSA, also known as lithium bis (trifluoromethanesulfonyl) imide, LiTFSI [29]) is mentioned to improve the cell safety additionally [25,30,31,32]. Unfortunately, electrolyte mixtures based on high-boiling solvents and LiTFSA exhibit unfavorable flow characteristics, which impede the use of those electrolytes in Li-ion-batteries in terms of cell performance and liquid wettability.

The aim of the study is to evaluate the method of drop shape analysis, which is used for investigating the electrolyte–separator interaction and is often linked to the wettability [5,12,33,34,35] as an appropriate tool for predicting the usability of electrolyte–separator couples for Li-ion batteries. Selected solvents with respect to safety and high voltage stability are chosen for this study. After presenting fundamental data about electrolytes and separators, the interaction between both materials and their use in Li-ion cells are presented and discussed. Additionally, the particle surface of a particle-coated PE separator is functionalized by silanization and the effect of such a surface modification in terms of surface polarity is investigated with respect to electrolyte interaction.

## 2. Results and Discussion

### 2.1. Electrolyte Mixtures

**Figure 1:**
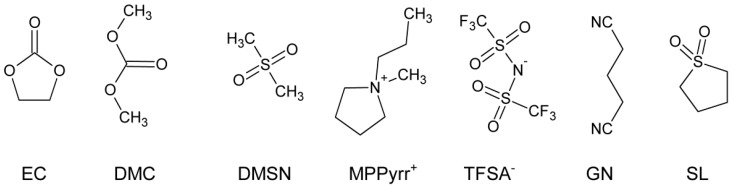
Chemical structures of ethylene carbonate (EC), dimethyl carbonate (DMC), dimethyl sulfone (DMSN), 3-methyl-1-propylpyrrolidinium bis (trifluoromethanesulfonyl) azanide (MPPyrr-TFSA), glutaronitrile (GN) and sulfolane (SL).

The chemical structures of the solvents, which are used in this study, are depicted in Figure 1. An overview of the electrolyte mixtures that are studied in the following sections is presented in Table 1. The solvents are chosen based on their safety characteristics (high flash points, see Table 2) and oxidative stabilities for high voltage applications [17,36]. The concentration, *c*, of the conducting salt (CS) is related to the total weight of electrolyte mixture (salt plus solvent).

**Table 1:** Composition of electrolyte mixtures.

Sample	Solvent	Ratio (wt:wt)	Conducting Salt (CS)	*c* (CS) ^[a]^ mol∙kg^−1^
**M-1**	EC/DMC	50:50	LiPF_6_	0.787
**M-2**	EC/DMSN ^[b]^	79.8:20	LiPF_6_	0.75
**M-3**	EC/DMSN ^[b]^	79.8:20	LiTFSA	0.75
**M-4**	MPPyrr-TFSA	100	LiTFSA	0.75
**M-5**	GN	100	LiTFSA	0.75
**M-6**	SL	100	LiTFSA	0.75

^[a]^ CS = conducting salt; weighted portion in the electrolyte mixture; ^[b]^ 0.2 wt % hexamethyldisilazane (HMDS) was added with respect to the solvent mixture to ensure a water-free electrolyte. Additionally, HMDS is able to sequester traces of HF.

### 2.2. Physicochemical Properties

In Table 2, selected properties of the electrolytes are provided. The surface tensions of the electrolyte mixtures are measured according to the pendant drop method. In the literature, a value of the surface tension of electrolyte mixture M-1 is mentioned at γ_L_ = 30.3 mN∙m^−1^ [34], which is in good correlation compared to the measurement here (γ_L_ = 29.4 ± 0.2 mN∙m^−1^). The surface tension of the different electrolytes covers the range of γ_L_ = (23.3–38.1) mN∙m^−1^.

**Table 2:** Physical properties of electrolyte mixtures: *T*_c_, crystallizing temperature; *T*_m_, melting point; fp, flash point; *d*, density; η, viscosity; κ, conductivity, *E*_ox_, electrochemical oxidative stability (against Pt); and γ_L_, surface tension.

Sample	*T*_c_/°C (DSC, onset) ^[a]^	*T*_m_/°C (DSC, peak max.) ^[a]^	*D* (25 °C) g∙cm^−3^ ±0.0005	η (20 °C) mPa∙s	κ ^[b]^ (20 °C) mS∙cm^−1^ (*n* = 3)	γ_L_^[b]^ (25 ± 2 °C) mN∙m^−1^ (*n* = 25)	*E*_ox_ (5 mV∙s^−1^) V *vs.* Li/Li^+^ (±0.1 V)	fp ^[c]^ (°C)
**M-1**	−39.4	−20.5	1.3030	4.4 ± 0.1	10.3 ± 0.5	29.4 ± 0.2	5.6	24
**M-2**	−20.1	14.6	1.3996	12.1 ± 0.2	6.1 ± 0.3	38.1 ± 0.3	5.6	142
**M-3**	−39.4	12.4	1.4260	13.4 ± 0.2	5.3 ± 0.2	34.3 ± 0.1	5.4	142
**M-4**	−12.1	27.1	1.5210	399.8 ± 0.8	0.53 ± 0.05	23.3 ± 0.2	5.5	353 ^[d]^
**M-5**	−42.3 ^[e]^	−30.4	1.1082	19.0 ± 0.2	2.4 ± 0.1	32.9 ± 0.1	5.6	112 ^[f]^
**M-6**	−59.3	−8.0	1.3760	37.8 ± 0.2	2.1 ± 0.1	33.1 ± 0.4	5.0	151

^[a]^ at 10 K·min^−1^; ^[b]^ Provided is the value of the standard deviation (SD) of *n* individual measurements; ^[c]^ Provided is the flash point of the pure solvent otherwise mentioned; ^[d]^ Flash point of 1M LiTFSA in MPPyrr-TFSA; ^[e]^ recrystallization at heating; peak maximum and ^[f]^ Data taken from www.merckmillipore.com.

**Figure 2:**
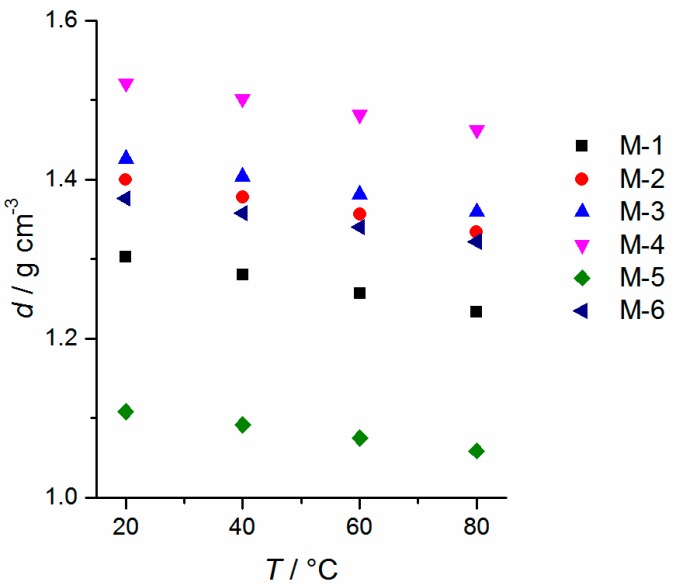
Temperature-dependent values of the density of mixtures M-*n* (*n* = 1–6).

**Figure 3:**
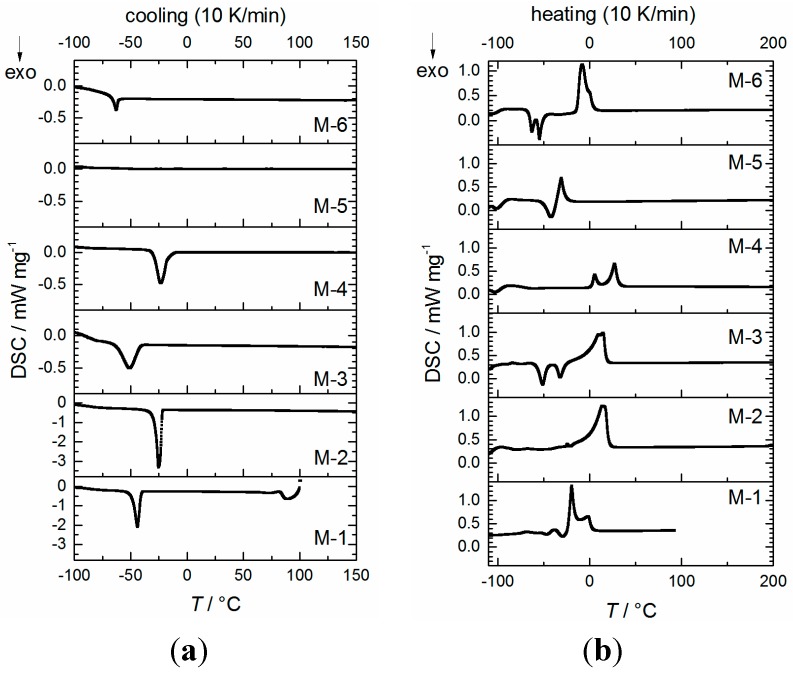
DSC measurements of mixtures M-*n* (*n* = 1–6) in closed cup during cooling (**a**) and heating (**b**) at 10 K·min^−1^ (*exo* down).

**Figure 4:**
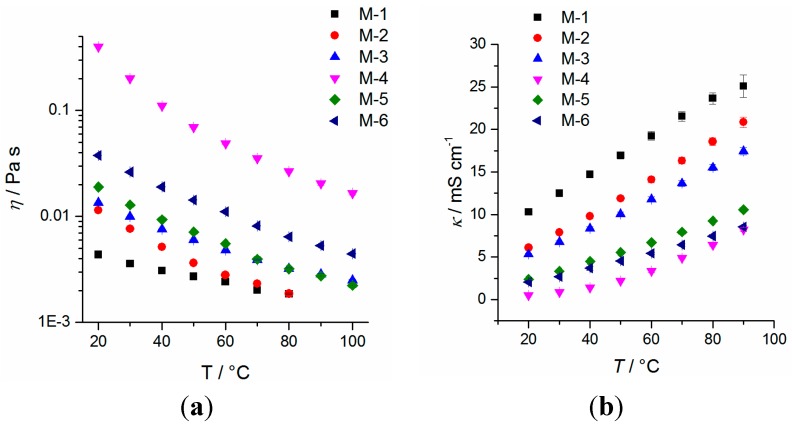
Temperature-dependent values of the viscosity (**a**) and conductivity (**b**) of mixtures M-*n* (*n* = 1–6) between 20–100 °C (viscosity) and 20–90 °C (conductivity). See Table S3 for detailed values.

The oxidative stability of the electrolytes was measured against Pt working electrodes. The measurements reveal an excellent oxidative stability of >5 V *vs.* Li/Li^+^ for all electrolyte mixtures (a detailed diagram is shown in supporting information (Figure S1)). Therefore, all electrolyte mixtures should withstand cell voltages up to 4.2 V *vs.* Li/Li^+^ and therefore should be suitable as electrolytes for NMC-based Li-ion cells based on its potential window. All mixtures are in liquid state at room temperature and the melting point is depressed compared to pure solvents when LiTFSA is added. It should be mentioned that the melting point is better described by the onset than the peak maximum in DSC measurements, albeit the determination of the onset is complicated by solid–solid transitions or recrystallizations. The temperature dependency of the density values is depicted in Figure 2, where the lowest value is received for the nitrile based electrolyte M-5 and the highest value for ionic liquid based mixture M-4. The temperature dependence for all mixtures is in similar order of magnitude, which can be quantified by the quotient *d*_20 °C_/*d*_80 °C_ = 1.047 ± 0.006 for all mixtures. DSC measurements of the electrolyte mixtures are depicted in Figure 3. The maximum temperature of mixture M-1 during the measurement is set to 100 °C because of the low boiling point component dimethyl carbonate, whereas the other mixtures are investigated up to 200 °C (closed cup). It is supposed that within a cooling rate of 5 to 20 K∙min^−1^ the formation of a non-crystalline phase in case of mixture M-5 is favored, thus no crystallizing point can be detected. In this case, a distinct recrystallization during heating is found. Electrolytes M-3 and M-6 exhibit a recrystallization (Figure 3b, *exo*-peak) during heating as well, when the cooling is performed at 5 to 20 K∙min^−1^. It should be noted that the appearance of exothermal features in the DSC trace of sample M-1, M-3, M-5 and M-6 indicate the presence of amorphous phases. The DSC measurements reveal that all electrolyte mixtures are in liquid state at room temperature. Temperature-dependent viscosity and conductivity measurements are shown in Figure 4 for all mixtures. The detailed values are listed in Table S1.

It can be observed that the temperature dependence of the viscosity is at a maximum for mixture M-4 (*η*_20 °C_/*η*_80 °C_ = 15; Table S2), which can be explained based on the strong attractive interactions of the ionic liquid and LiTFSA. All viscosity values are in a range of η = 4–400 mPa∙s at 20 °C, whereas the conductivity of the electrolyte is in the range between 0.5–10.5 mS∙cm^−1^. It is observed that the conductivity of all mixtures with the exception of ionic liquid mixture M-4 is >1 mS∙cm^−1^, which is said to be a requirement for the use as liquid electrolyte in Li-ion batteries [37]. Therefore, Li-ion cell testing of electrolyte M-4 is expected to result in poor cell performance only. It can be shown that fitting of the viscosity data according to Vogel-Fulcher-Tammann-Hesse (VFTH) can be done within *R*^2^ ≥ 0.995 and that the Walden rule (κ~η^−1^) is fulfilled within *R*^2^ ≥ 0.989. Fitting data and parameters are listed in Table S1 and Table S2 in the supporting information. Principally the measurement results reveal that based on physicochemical properties all mixtures are expected to be suitable for Li-ion cells at least at small discharging C-rates.

### 2.3. Separators and SEM Imaging

**Figure 5:**
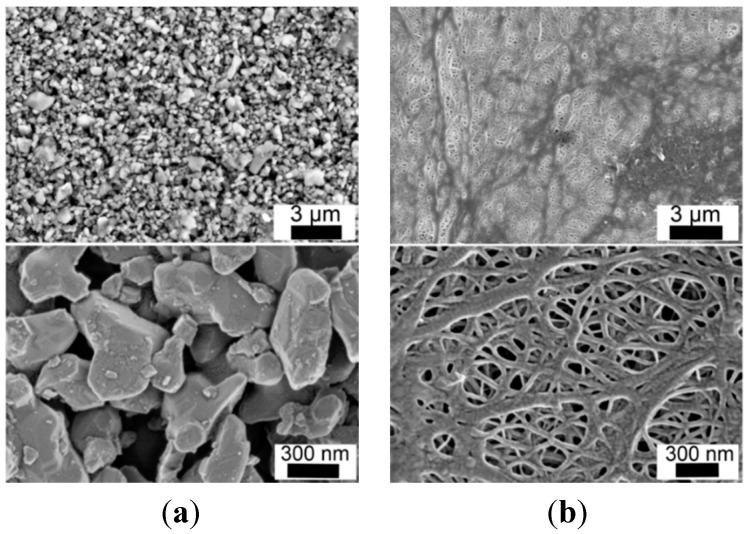
Scanning electron microscopy (SEM) picture of the surface of separator COD-20 (**a**) and COATED (**b**).

**Figure 6:**
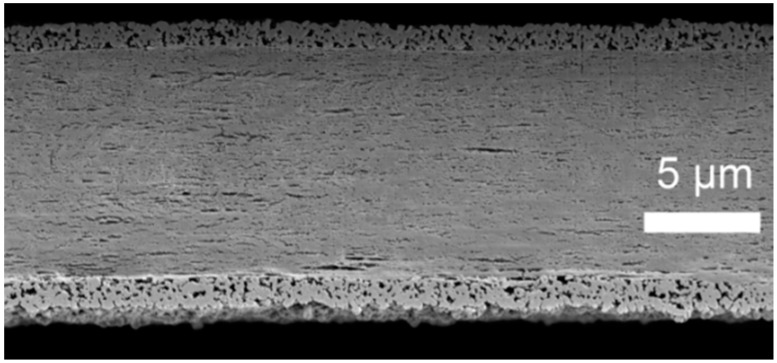
SEM picture of the cross section of separator COATED prepared by ion beam cutting.

For this study, a polyethylene (PE) separator (COD-20) and a particle-coated PE separator (COATED) are used in comparison. Main characteristics are summarized in Table 3. The specific surface of both separators was measured (Table 3), where it differs by a factor of ~2 based on the additional weight of the ceramic layer. Both commercial separators are analyzed via scanning electron microscope (SEM) to visualize their surface structure (Figure 5). The cross section of the separator COATED is depicted in Figure 6. Both separators are composed of a polyethylene based porous membrane with its typical porous structure (Figure 5a). Based on the SEM images, the pore sizes can be estimated to be ~100–200 nm, which is much larger (~factor 100–500) than any hydrodynamic diameter of cationic Li^+^ species or anionic salts that are discussed in literature [38,39,40]. The separator COATED is covered by irregular shaped particles (size ≤ 2 µm) on its surface (Figure 5b), which are mainly identified as Al_2_O_3_ particles in energy dispersive X-ray spectroscopy (EDS) analysis. Traces of the elements Na and C were also observed, among others, on the surface of separator COATED, which can be explained by impurities and organic binder compounds. In the cross section image (separator COATED, Figure 6), it is seen that the surface of the porous PE membrane is completely covered by a porous particle coating with a thickness of 2–3 µm on each side. The permeability of air is almost identical for both separators, which reveals that the coating does not affect the gas permeability. It should be mentioned that PE melts at ~137 °C (provided as shut-down temperature), which influences the separator characteristics severely. The temperatures, which are applied during drying procedures, are therefore carefully controlled and not exceeded above 80 °C to ensure not to destroy the porosity fine structure.

**Table 3:** Both separators and selected properties based on supplier information.

Separator	Thickness ^[a]^ µm	Permeability ^[a,b]^ s/100 mL	Porosity ^[a,c]^ %	Heat Shrinkage ^[c]^ % at 105 °C, 1 h	Specific Surface m^2^∙g^−1^
**COD-20**	19.1	237.6	42.3	2.4	42.4
**COATED**	20.6	241.0	n/a ^[d]^	1.5	23.7

^[a]^ Values are provided by supplier; ^[b]^ Gurley type; ^[c]^ calculated value; and ^[d]^ n/a = not available.

### 2.4. Contact Angle Measurements and Drop Shape Analysis of the Separators

Contact angles can be used as a measure of the interaction of liquids with surfaces. Principally, the equilibrium contact angle is defined as the angle between the liquid/gas interface and the solid surface, it quantifies the wettability of the solid surface by the liquid and it reflects the strength of the molecular interactions of liquid, solid and vapor. In the case of porous materials, the measurement of contact angles is complicated by the uptake of the solvent from the porous materials. In the case of Li-ion batteries, the microscopic surface of the separator (porous materials) is obviously not smooth and the surface fine structure (roughness) is small compared to the drop area. Thus, the macroscopic observed contact angle is different from the microscopic contact angle between the separator materials and liquid. Finally, a contact angle can only be observed on the surface of the material, thus a multi-layer buildup of materials allows only the study of the top layer. These factors cause a time-dependency of the contact angle in case of separator materials, where the evaporation of low boiling solvent components with time additionally hampers the analysis (volume of droplet = 1–4 µL). The surface of a separator is a very critical parameter within the contact angle analysis, thus same conditions are a prerequisite for comparable measurement results. In the study, all separators were dried carefully (75 °C, 24 h, 1 mbar) and stored afterwards in closed boxes inside a glove box (O_2_/H_2_O < 0.5 ppm). The measurement was performed inside a glove box (dry air, H_2_O < 20 ppm) as well to ensure not to contaminate the separator surface by water traces (especially important in case of ceramic particles onto the surface).

**Figure 7:**
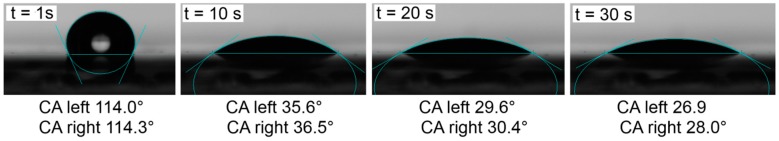
Time-dependency of the contact angle between separator COATED and mixture M-2 as a representative for all electrolyte mixtures and both separators.

**Figure 8:**
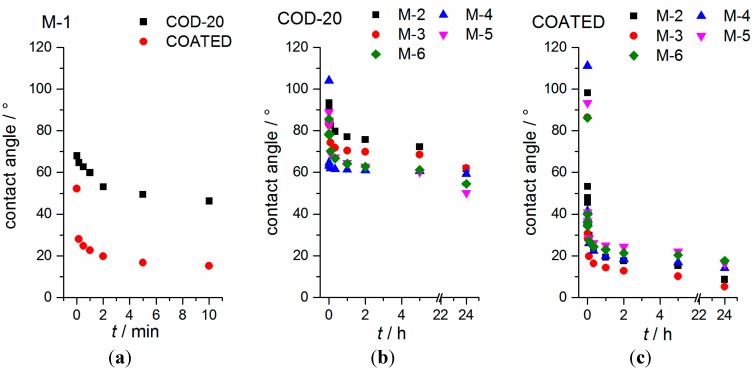
Time-dependent contact angles between separator and mixtures. (**a**) Contact angles between mixture M-1 and both separators are depicted within 10 min; (**b**) Contact angles between mixture M-*n* (*n* = 2–6) and separator COD-20 are shown within 24 h; and (**c**) Contact angles between mixture M-*n* (*n* = 2–6) and separator COATED are compared within 24 h.

A representative behavior of the time-dependency of the contact angle is depicted in Figure 7 within *t* = 30 s. Principally, each droplet results in two contact angles, whereas at least 10 droplets are studied for all combination possibilities. It should be mentioned that the volume of the drop is large compared to the possible liquid uptake from the separator based on its thickness, thus a complete uptake into the interior of the separator is excluded. In Figure 8, the interaction of mixture M-1 with both separators is depicted within *t* = 10 min. The time period is chosen as a compromise between the evaporation of DMC and the relaxation time of forming a stable contact angle. A distinct difference can be observed between separator COD-20 (θ = 46.2° ± 1.1°) and COATED (θ = 15.2° ± 1.8°) in terms of the contact angle 10 min after dropping, which is significantly reduced by factor 3 when the PE polymer is coated by ceramic particles. A similar behavior is found for all other mixtures likewise (Figure 8). It demonstrates that polar ceramic particles combined with an increased surface roughness (taken from Figure 5) reduce the contact angle between separator and liquid phase significantly. Furthermore, it can be seen, that this effect is almost independent of surface tensions of the electrolytes (γ_L_ = 23.3–38.1 mN·m^−1^). It is observed that the reduction of contact angles between 5 and 24 h, where the separators are stored uncovered inside a glove box, is more pronounced in the case of separator COATED (factor 1.2–1.9) than of separator COD-20 (factor 1.0–1.2).

**Figure 9:**
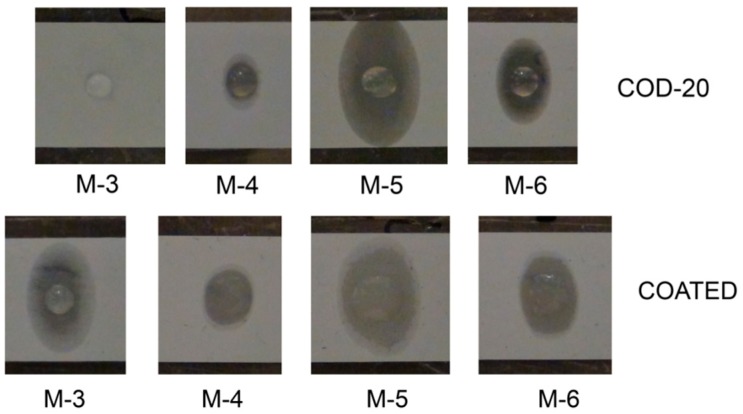
Representative photographs of drops onto separator foils 5 h after dropping (inside glove box) with selected mixtures: (**Above**) separator COD-20; and (**Below**) separator COATED.

The shape of the drops after 5 h is depicted in Figure 9 exemplarily for mixture M-3, M-4, M-5 and M-6. It can be seen that differences in the wetting characteristics result even when contact angles are in the same order of magnitude (e.g., COD-20, M-3 *vs.* M-5). This finding already demonstrates that the contact angle alone is not sufficient for a comprehensive characterization of separator–electrolyte interactions, nor as proof of appropriate electrolyte–separator usability. Principally, it is difficult to distinguish the uptake into the interior of the separator from the spreading of the liquid in the top surface layer of the separator based on both experimental techniques (contact angle and drop visualization). Multi-layer separators additionally impede such an analysis. Nevertheless, based on these results one could suggest a liquid uptake in all cases (eventually with exception of mixture M-2/M-3 + COD-20) and a more readily uptake in case of the separator COATED.

### 2.5. Cell Cycling (NMC|Li Coin Cells) and Separator Penetration

The principal applicability of the mixtures as Li-ion electrolytes is investigated in CR 2032 coin cells in Li|NMC configuration (*E* = 3–4.2 V *vs.* Li/Li^+^). The half-cell geometry was chosen to exclude that the electrolytes are inoperative as a result of graphite interactions (e.g., exfoliation, insufficient solid electrolyte interface), which necessitate the use of additional additives like vinylene carbonate or lithium bis (oxalato) borate. In those cases, the wetting characteristics of the separators could be modified by these additives. Furthermore, a low C-Rate (C/25) is used (during charging and discharging) at the beginning to ensure a proper Li^+^ transport even in case of ionic liquid mixture M-4 (maximum C-rate = 1C). Obviously, a low charging and discharging rate favors the formation of Li-dendrites, particularly during the charging cycle. Nevertheless, it is expected that the separator will protect both electrodes from Li-dendritic short circuits for at least a few cycles if the Li^+^ transport is getting through the separator.

**Figure 10:**
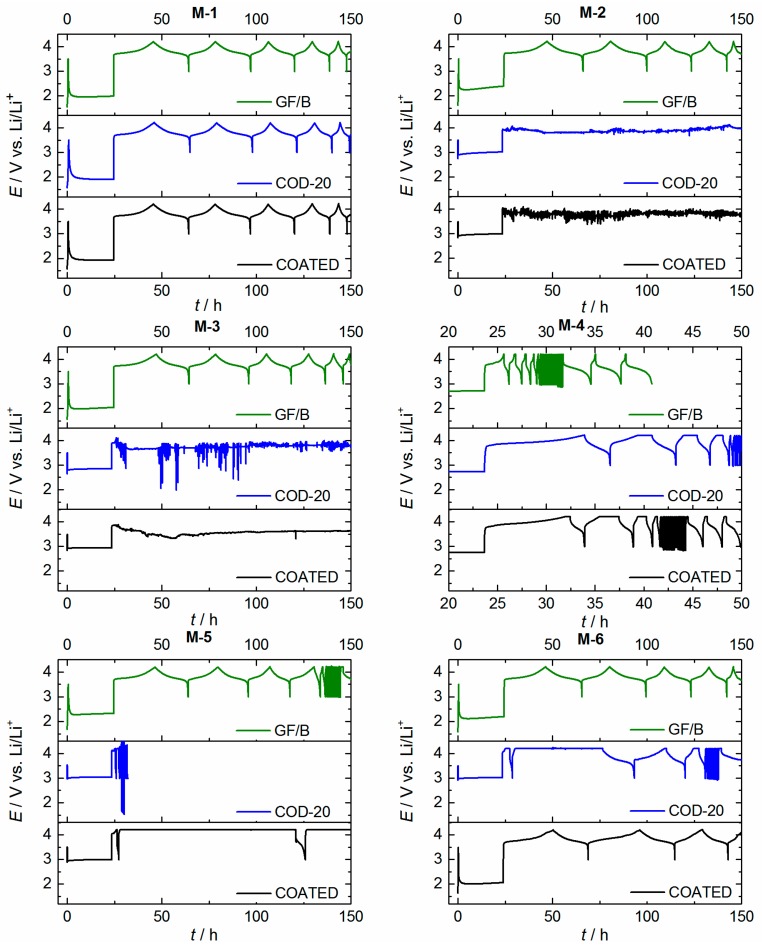
Cell cycling of NMC|Li (NMC = LiNi_1/3_Mn_1/3_Co_1/3_O_2_) half cells with separator GF/B, COD-20 and COATED for each electrolyte mixture without additional additives. After a short-term jump charge (3.5 V *vs.* Li/Li^+^), the cells are rested at open circuit for 24 h. Afterwards, the cells are cycled between 3–4.2 V *vs.* Li/Li ^+^. Parameters: Coin cells CR 2032, *T* = 25 ± 1 °C, active mass (NMC) = 12.2 ± 0.5 mg∙cm^−2^, *d* (separator) =17 mm, *d* (Li, NMC) = 16 mm. All separators were soaked in the electrolyte for 4 h additionally.

Figure 10 summarizes the first 150 h of cycling. To ensure the principal working of the electrolytes, a glass fiber separator (Whatman, GF/B, thickness: 675 µm) is used for all mixtures in comparison. In case of glass fiber separators, all coin cells exhibit typical NMC charging and discharging characteristics which are expected from the materials. This supports that the mixtures in principle can be used as electrolytes and a cell cycling (at least at small C-rates) is observed in all cases. It should be noted that the use of highly viscous electrolyte mixtures hampers the mobility of Li-ions so much that the performance at fast C-rates is limited. In detail, the poor performance of ionic liquid electrolyte M-4 can be explained by a poor lithium transport [21]. It can be observed that the nitrile-based mixture M-5 is unusable without any additives at faster C-rates (C/2) likewise. Surprisingly, the cell performances of the coin cells that contain PE-based separators are completely different for most of the electrolyte mixtures. Detailed values about the specific capacity of NMC and the cycling performance of the cells within the first 5 cycles up to a discharge rate of C/5 are provided in Figure S2 and Table S4.

A fairly sufficient cell operating can only be observed in case of mixture M-1 (COD-20, COATED), M-4 (COD-20, COATED) and M-6 (COATED). Mixture M-4 combined with PE-based separators outperforms the cell performance against glass fiber separator GF/B because of the reduced thickness of those separators. For other mixtures and separator combinations (COATED, COD-20), the findings are characteristic for Li dendritic growth and indicate almost no Li transport from NMC to the Li surface. Little Li^+^ transport and consequentially a marginal cell operating can be observed for mixture M-6 and separator COD-20. These findings can neither be explained from the contact angle measurements (Figure 8) and surface wetting photographs (Figure 9) nor based on electrochemical properties from the mixtures. It should be mentioned that the flow characteristics (viscosity) of mixture M-4 and M-6 are inferior compared to mixture M-2, M-3 and M-5. It is supposed that the PE separator structure by itself principally impedes the liquid uptake based on polarity and porosity factors. To prove this assumption, the penetration of the liquid through the separator is investigated more carefully.

**Figure 11:**
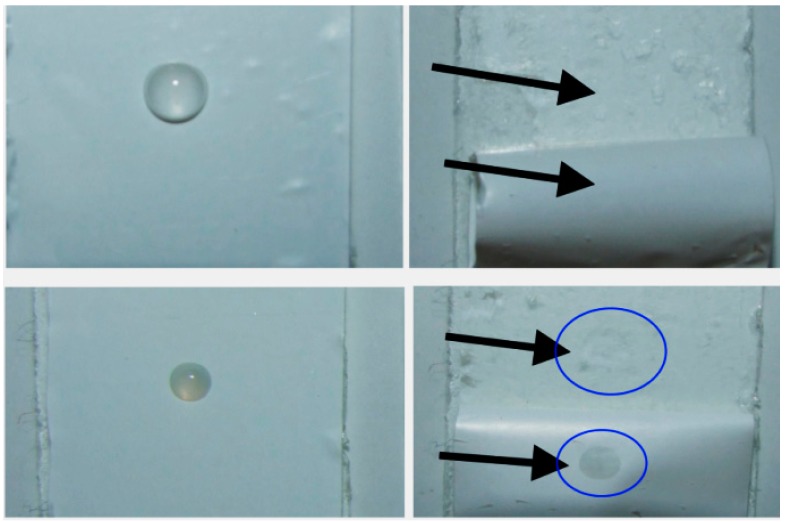
Backside wetting of separator COD-20 by two selected mixtures before (**left**) and after (**right**) pulling the separator off the tape. **Above**: No wetting is observed in case of mixture M-2; **Below**: Wetting of the backside can be observed in case of mixture M-1 (*t* = 5 min). Arrows and circles are painted for a better illustration of the drops.

It is possible to evaluate the solvent penetration with an easy method described as “backside wetting”. Here, a small solvent droplet is dropped in the middle of the separator, which is fixed via a tape onto a microscope slide, and after a certain time, *t*, the separator is pulled off the tape. If the liquid penetrates the separator, the tape on the backside will be wetted by the liquid, which can be observed easily. Figure 11 illustrates exemplarily two different observations (mixture M-1 and M-2 with separator COD-20) and the results of all electrolyte–separator couples are listed in Table 4. It is seen, that a liquid penetration is found in exactly the same cases as coin cells operate properly. During the measurements, no additional vacuum is applied. Additionally, no significant differences in the contact angle characteristics are obtained, when the surface of the separator COATED is functionalized with silane derivatives. Two selected silane derivatives, namely (2-cyanoethyl)triethoxysilane and *n*-propyltriethoxasilane, are used to slightly change the surface polarity and to affect the interactions between lithium ions and the separator surface. It should be noted that such a procedure only affects the ceramic particle layer; polyethylene cannot easily be functionalized with silane derivatives based on its chemical structure and reactivity. Therefore, the idea is to slightly modify the upper ceramic layer so much that the solvent spreads more easily within the ceramic coating and wets the PE layer on a larger area. The contact angles of these separators are slightly enhanced to those of separator COATED as expected from polar–polar interactions (Figure S3). Nevertheless, the penetration studied by backside wetting of the electrolytes into these functionalized separators are comparable to the findings of separator COATED, which reveal that the silanization of the separator only affect the ceramic particles layer.

At least two reasons can cause such an behavior: (1) Although all separators are soaked in the electrolytes and the cells are additionally equilibrated for 24 h for an liquid uptake in NMC and separator materials, this is still not sufficient for wetting the separators in the interior (thickness of both PE of <21 µm); (2) Despite sufficient solvent wetting, the Li^+^ ions are not able to move through the separator due to bulky Li^+^-solvent complexes. It is expected and already described in literature [41,42,43,44,45,46] that the ionic liquids will strongly interact with Li^+^ ions.

**Table 4:** Penetration through the separator ^[a]^.

Sample	COD-20	COATED	COATED, Silanized ^[b]^
**M-1**	++	++	++
**M-2**	−	−	−
**M-3**	−	−	−
**M-4**	++	++	++
**M-5**	−	−	−
**M-6**	−	o	+

^[a]^ “++” backside wetting immediately after application of a droplet (< 1 min); “+” entire backside wetting within 5 min; “o” poor backside wetting even after 10 min; “−“ no backside wetting after 15 min; ^[b]^ Silanization of the separator COATED with propyltrimethoxysilane or 2-cyanoethyltrimethoxysilane.

These associations are supposed to form more bulky complexes than those complexes composed of Li^+^ and organic molecules. However, mixture M-4 enables better cycling characteristics than organic solvents based electrolytes. Therefore, it is expected that the wetting inside the separator caused failure of cell cycling. It should be noted that liquid uptake is studied, *inter alia*, by time-dependent weighing of separators, which are placed in electrolyte solution [47]. Nevertheless is should be mentioned that it is for one thing difficult to determine the difference in weight with and without solvent for very thin (<20 µm) separators (results are fraught with high uncertainty). In addition, liquid uptake from different layers (coating and polyolefin membrane) is almost indiscernible from each other. Principally, the liquid uptake can be enhanced by vacuum during electrolyte filling, which was not investigated in this study.

## 3. Experimental Section

Ethylene carbonate, sulfolane, glutaronitrile, 1-methyl-3-propylpyrrolidinium bis (trifluoromethane-sulfonyl) azanide and LiTFSA (Iolitec, 99%) were dried by using a coulombmetric Karl-Fischer titrator, which consists of a 831 KF Coulometer and a 860 KF Thermoprep oven from Metrohm (sample in extra oven, dry gas flow, 130 °C). Dimethyl sulfone was sublimated by using a Büchi oven at 150–155 °C (2.1 mbar). In Table 5, the solvents are summarized and selected properties (melting point, boiling point) are provided. LiPF_6_ (Sigma–Aldrich, 99.99%, anhydrous, Taufkirchen, Germany) and mixture M-1 (EC/DMC + 1M LiPF_6_; Aldrich; battery grade) were used without purification. The preparation of the electrolytes was performed in an argon-filled glove box (MBraun GmbH, Garching, Germany) with oxygen and water levels below 0.5 ppm. NMC electrodes were prepared by standard casting techniques using NMC (LiNi_1/3_Mn_1/3_Co_1/3_O_2_, 92 wt %, NM-3100, Toda America, Battle Creek, MI, USA), carbon black (4 wt %, Super C65, Imerys, Graphite & Carbon, Bodio, Switzerland), PVDF (4 wt %, Solef 5130) and NMP (Sigma–Aldrich, Taufkirchen, Germany) [48].

The separators COD-20 (PE, 19.1 µm thick) and COATED (PE, 20.6 µm thick with particle coating) from Targray (both model names) were used. For silanization, the separators were placed in a mixture of (2-cyanoethyl) triethoxysilane (97%, Alfa Aesar, Karlsruhe, Germany) or *n*-propyltriethoxysilane and dry ethanol (10 vol % silane) for 60 min and washed five times with pure anhydrous ethanol. Subsequently, the separators were dried at 80 °C for 2 h.

**Table 5:** Purity and selected properties (mp = melting point, bp = boiling point) of chemicals, which are used in the study (pure solvents, preparation of electrolyte mixtures afterwards).

Solvent ^[a]^	mp^[b]^/°C (Solvent)	bp^[b]^/°C (Solvent)	Supplier	Purity	Purification for This Study
**EC**	35–37	244–245	Sigma-Aldrich	99% anhydrous	drying ^[c]^
**DMSN**	108–10	238	Alfa Aesar	99%	sublimation ^[d]^
**MPPyrr-TFSA**	12	NN	Iolitec	99%	drying ^[c]^
**GN**	−29	286–288	Aldrich	99%	drying ^[c]^
**SL**	25–28	285	Aldrich	99%	drying ^[c]^

^[a]^ EC = ethylene carbonate; DMSN = dimethyl sulfone; MPPrr = 1-methyl-1-propylpyrrolidinium; TFSA = bis (trifluoromethanesulfonyl) azanide; GN = glutaronitrile; SL = sulfolane; ^[b]^ The data taken from chemspider.com; ^[c]^ Drying by using a Karl-Fischer-oven until no more water is detected caused by a voltage drift. ^[d]^ DMSN was sublimated (Büchi oven; 150–155 °C; 2.1 mbar) and not dried after the sublimation (handling under air).

In this study, coin cells (type: CR 2032, Hohsen, Chuo-ku, Osaka, JAPAN) were used with a coin cell crimper from BT Innovations. The cells were assembled in an argon-filled glove box according to standard procedures. Precisely, a lithium anode (Ø = 14 mm), a NMC cathode (Ø = 15 cm), and the separator (COD-20, COATED, Whatman^®^ GF/B) were used inside a coin type cell with one spring and one stainless steel spacer. The active material content of an electrode disk is ~23 mg.

The ionic conductivity, DSC and flash points were measured as described elsewhere [19]. The temperature range of the conductivity was restricted up to 90 °C because of the experimental setup.

Dynamic viscosity was measured using a Malvern Gemini HR Nano rotational rheometer with 40/1° cone geometry and a gap of 30 µm and 60/2° cone geometry and a gap of 150 µm. A temperature sweep between 20–100 °C as well as a shear rate sweep between 70–140 s^−1^ were performed and repeated at least three times. The mean value of these measurements is provided in the manuscript. These experiments were performed by using a solvent evaporation protecting cover in air.

The cyclovoltammograms were measured with a Zahner XPOT potentiostat (software: PPSeries, Potentiostat XPot Zahner elektrik 6.4, ZAHNER-elektrik I. Zahner-Schiller GmbH & Co. KG, Kronach-Gundelsdorf, Germany). The potential range was set to 3–6 V *vs.* Li/Li^+^ with platinum as the working electrode. The cells were measured in three-electrode configuration (EE-Cells manufactured by EL-Cell GmbH) with reference and working electrodes composed of lithium.

The density of selected mixtures was obtained by a precision densitometer from Anton Paar (DMA4500M) in a temperature range of 20–80 °C. The test cell was calibrated (Milli-Q water and air) and controlled against air. During the sample preparation in the measurement device, the mixtures were exposed to air for <5 s. The accuracy of the temperature was ±0.01 °C during the measurement.

The contact angles were measured according to the sessile drop method using an OCA 20 from DataPhysics Instruments GmbH. The device was placed in a glove box, which was filled (perfused) with dried compressed air (AD70L from Peak scientific was used for drying the compressed air). The water content in the box was determined by a dew transmitter (Easidew, Michell Instruments, Rowley, MA, USA, −100 to 20 °C (±2 °C)) from Michell Instruments to be less than −68 ± 2 °C (<20 ppm H_2_O). The separators (Targray, COATED and COD 20; PE-based) were fixed with tape (3 M, 50 µm; temperature stable up to 150 °C) onto a glass surface for the measurements, dried at 75 °C for 24 h in vacuum and stored afterwards in a closed glass jar inside the glove box. The drops were supplied by dosage needles (Nordson, 0.41 mm, 7018266, Nordson Deutschland GmbH, Erkrath, Germany). The temperature was detected to be 25–27 °C. At least 10 drops at different positions on the material were analyzed giving 20 values of the contact angle. The final value was determined by calculating the arithmetic mean and the standard deviation (SD; *n* = 20). All contact angles given in the manuscript are provided as value ± SD. Time-dependent measurements of each drop were performed up to 24 h after dropping. For the data processing of the resulting video file (first 30 s) and the pictures (after 30 s), the software SCA20 (v 3.7.4 build98) from DataPhysics Instruments was used (Data Physics GmbH, Bad Vilbel, Germany).

The surface tension was determined according to the pendant drop method on the device OCA 20 (DataPhysics, Data Physics GmbH, Bad Vilbel, Germany) inside a glove box (dew point of dry air = −68 ± 2 °C). A video of the drop was recorded during the continuous increase of the drop volume (dose rate = 0.1 µL∙s^−1^) until the drop was released. The last five sequence images (frames) before the drop is released were used to calculate the surface tension according to the Young-Laplace drop shape (needle: Gauge 15). The measurements were repeated five times (25 drops were analyzed) and the arithmetic mean including the standard deviation (SD) is provided in the text. The density of air (0.00129 g∙cm^−3^), the gravitational acceleration (9.8095 m∙s^−2^) and the density of the solvent mixture (see density measurements) are taken for the calculation.

The specific surface was obtained by the device Gemini VII 2390a Surface Analyzer from Micromeritics. The separators were dried at 65 °C under vacuum, nitrogen adsorption isotherms were measured and monomolecular capacities were calculated. Based on these results, specific surface values were derived from the monomolecular capacity based on a mean of the area of an absorbed nitrogen molecule in the monomolecular layer.

## 4. Conclusions

In this study, the interaction of selected electrolytes with polyethylene (PE)-based separators is investigated. Promising electrolyte mixtures for use in Li-ion batteries are chosen in terms of safety and high voltage stability characteristics and studied due to its fundamental physicochemical properties. The surface tension of the electrolyte mixtures are measured to be in the range of γ_L_ = 23.3–38.1 mN·m^−1^. Two separators composed of pure PE and ceramic-coated PE layer are chosen as representative for polyolefin separators and main characteristics of both separators are presented. The interaction between the separators and electrolyte mixtures is studied under dry conditions in terms of contact angles, liquid wetting and solvent penetration. The results are linked to cell tests in Li|NMC half cells which demonstrate a strong dependence of the cell operating from the separator–electrolyte interaction and the importance of the inner PE layer. High flash point electrolytes based on sulfolane or ionic liquids exhibit the best liquid penetration with a PE based separator. The best results are obtained with organic carbonates with low boiling point dimethyl carbonate and ethylene carbonate. It could be demonstrated that a ceramic coating is not sufficient to ensure entire liquid penetration into the interior of the separators. Furthermore, it is shown that the method of drop shape analysis can only be used in a very limited manner to predict the electrolyte uptake from multi-layer separators. However, a novel method called “backside wetting” is presented as an easy tool to evaluate the compatibility between novel separators and electrolytes.

## Supplementary Materials

Supplementary materials can be found at http://www.mdpi.com/1422-0067/16/09/20258/s1.
