# Supplementary material for: Interaction of High Flash Point Electrolytes and PE-Based Separators for Li-Ion Batteries
Source: Int J Mol Sci. 2015 Aug 27;16(9):20258–76. doi: 10.3390/ijms160920258 (PMC4613202; doi:10.3390/ijms160920258)
Supplement: Supplementary file 1 [file ijms-16-20258-s001.pdf]

Supplementary Information

Table S1. Free VFTH fitting of the viscosity data. For the fitting procedure, the following initial fitting parameters are used: η_0 = 0.2 mPa·s; D = 4; T_0 = 160 K. No additional assumptions were done during the fitting procedure.

Mixture	η_0 10^{-2} mPa s	T_0/K	D± (≤0.1)	m	T_g/K DSC *	R^2	$\eta_{20\,°C}/\eta_{80\,°C}$
M-1	13.4	107	6.0	114	– [a]	0.995	2.3
M-2	4.1	171	4.0	162	– [a]	0.999	6.1
M-3	13.0	153	4.2	154	189	0.999	4.2
M-4	56.6	209	2.6	240	217	0.999	15.0
M-5	2.8	128	8.4	86.2	169	0.999	5.9
M-6	3.7	112	11.3	68.3	– [a]	0.999	5.9

* DSC: heating at 10 K/min; [a] no T_g could be extracted from the measurement.

Table S2. Results of Walden fitting procedures.

Sample	$c_{c.s}$ [a]/mol·dm^{-3}(30 °C)	Slope	R^2
M-1	1.016	0.92 ± 0.03	0.992
M-2	1.043	0.83 ± 0.02	0.996
M-3	1.061	0.79 ± 0.01	0.999
M-4	1.132 [b]	0.95 ± 0.04	0.990
M-5	0.832	0.79 ± 0.03	0.989
M-6	1.023	0.76 ± 0.03	0.990

[a] It is obvious that the differences between the concentrations of the mixtures result from differences in densities; and [b] The total concentration of ions (LiTFSA + MPPyrr-TFSA) is 4.036 mol·dm^{-3} at 30 °C.

Table S3. Measured values of the conductivity κ and viscosity η of mixtures M-n (n = 1–6).

Sample	T/°C	κ [a]/mS cm^{-1}	η [b]/mPa s
M-1	20	10.3 ± 0.3	4.4 ± 0.1
M-2	20	6.1 ± 0.1	11.4 ± 0.1
M-3	20	5.3 ± 0.1	13.4 ± 0.1
M-4	20	0.53 ± 0.01	399.7 ± 0.8
M-5	20	2.4 ± 0.1	19.0 ± 0.2
M-6	20	2.1 ± 0.1	37.8 ± 0.1
M-1	40	14.7 ± 0.4	3.1 ± 0.1
M-2	40	9.8 ± 0.2	5.1 ± 0.1
M-3	40	8.4 ± 0.2	7.6 ± 0.1
M-4	40	1.4 ± 0.1	111.0 ± 1.0
M-5	40	4.5 ± 0.1	9.3 ± 0.1
M-6	40	3.7 ± 0.2	19.1 ± 0.1
M-1	60	19.2 ± 0.5	2.42 ± 0.03
M-2	60	14.1 ± 0.3	2.8 ± 0.1
M-3	60	11.8 ± 0.3	4.8 ± 0.1
M-4	60	3.4 ± 0.2	48.8 ± 0.9
M-5	60	6.7 ± 0.2	5.5 ± 0.1
M-6	60	5.4 ± 0.1	11.1 ± 0.1

Table S3. *Cont.*

Sample	*T*/°C	κ [a]/mS cm^{-1}	η [b]/mPa s
M-1	80	23.7 ± 0.7	1.86 ± 0.03
M-2	80	18.6 ± 0.5	1.87 ± 0.05
M-3	80	15.5 ± 0.4	3.2 ± 0.1
M-4	80	6.5 ± 0.2	26.7 ± 0.7
M-5	80	9.2 ± 0.2	3.2 ± 0.1
M-6	80	7.5 ± 0.2	6.4 ± 0.1

[a] Provided is the standard error (SE) from three individual measurements; and [b] Provided is the standard error of 25 data values which are taken at shear rates between 70–140 s^{-1} from three individual measurements.

Table S4. Specific capacities NMC|Li cells at room temperature based on cell performance tests at cycle 1 (C = charging, DC = discharging) and cycle 4.

Cycle [a]	Separator	M-1	M-2	M-3	M-4	M-5	M-6
C-1	GF/B	171.6	181.7	185.2	11.3	177.9	177.4
DC-1	GF/B	149.3	152.0	137.3	3.9	139.4	154.4
C-4	GF/B	148.5	150.4	146.2	5.6	204.6	153.4
DC-4	GF/B	146.5	149.2	139.7	4.3	50.1	148.5
C-1	COD-20	174.9	1327.8	1313.3	77.3	8.5	20.9
DC-1	COD-20	152.1	–	–	20.7	4.1	11.6
C-4	COD-20	149.1	–	–	17.1	0.9	129.3
DC-4	COD-20	147.6	–	–	9.7	0.1	52.5
C-1	COATED	172.5	1329.4	1323	64.7	15.5	209.6
DC-1	COATED	150.2	–	–	11.6	8.0	147.7
C-4	COATED	150.1	–	–	6.6	–	173.2
DC-4	COATED	148.7	–	–	3.9	–	145.4

[a] C-rates: C-1: C/30; DC-1: C/25; C-4: C/10; DC-4: C/10.

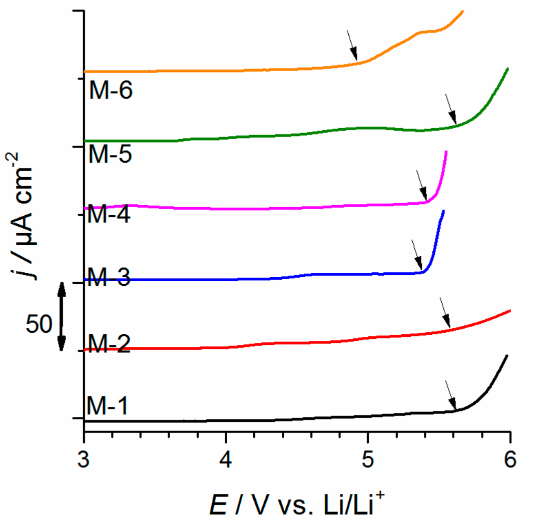

Figure S1. Current density response of electrolyte mixtures in Pt|Li cell configuration at room temperature and $v = 1$ mV·s^{-1}. The arrows indicate the oxidative stability limit based on a tangent procedure.

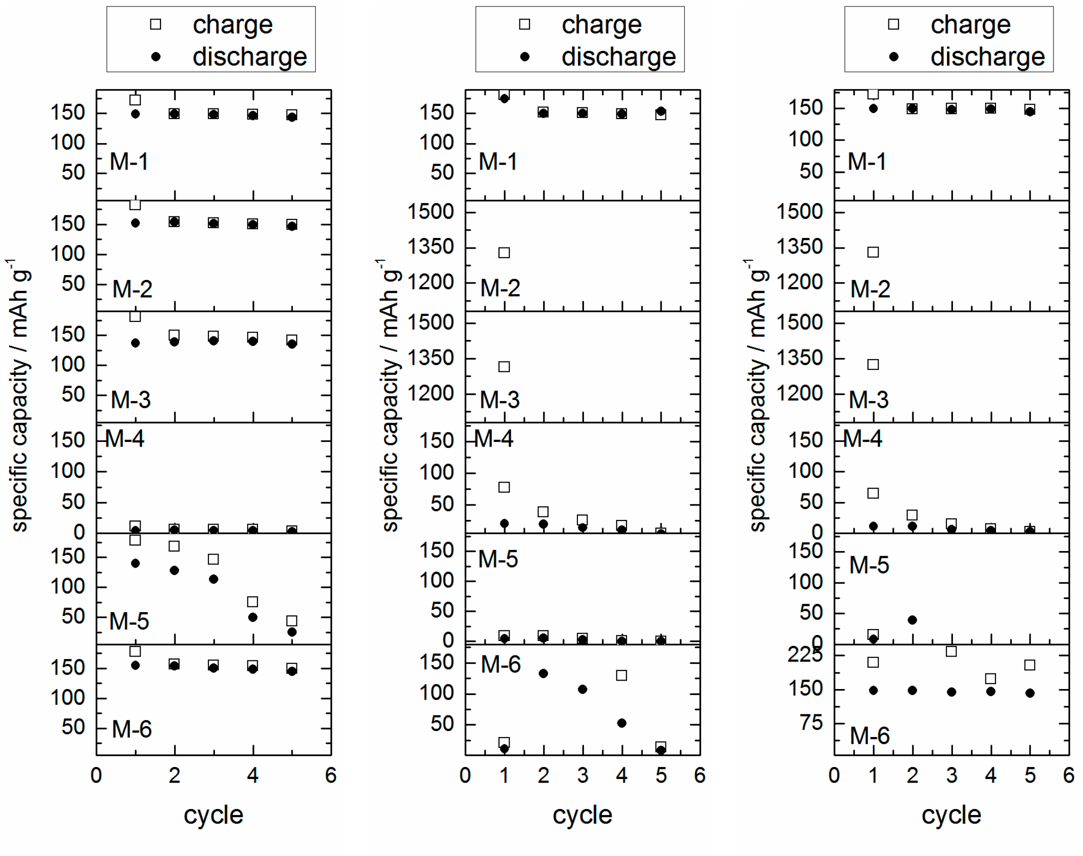

(**a**) (**b**) (**c**)

Figure S2. Charge and discharge capacity of electrolyte mixture investigated in the study. (**a**) GF/B separator; (**b**) COD separator; (**c**) COATED separator. Cell configuration: Li|NMC. square = charging, circle = discharging. C-rate (charging–discharging): cycle 1: C/30–C/25; cycle 2: C/15–C/25; cycle 3: C/10–C/15; cycle 4: C/10–C/10; cycle 5: C/5–C/5.

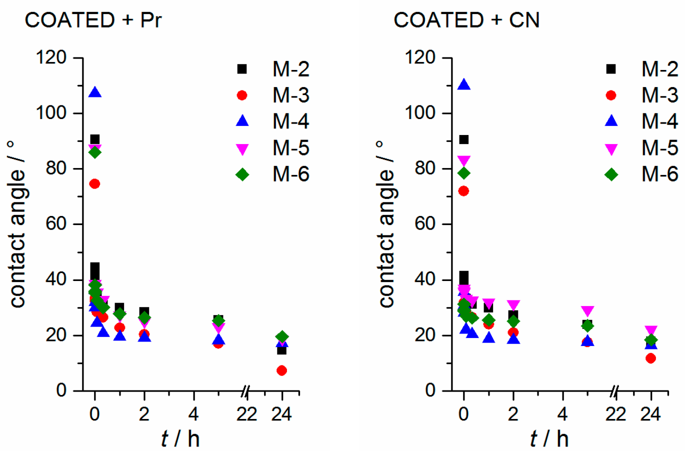

(**a**) (**b**)

Figure S3. Time-dependent contact angles between silane functionalized separators and mixtures. (**a**) Contact angles between mixture M-*n* (*n* = 2–6) and propyl-functionalized separator COATED + Pr are shown within 24 h; and (**b**) Contact angles between mixture M-*n* (*n* = 2–6) and cyano-functionalized separator COATED + CN are compared within 24 h.
